# The Corset, a Cause of Consumption

**Published:** 1889-07

**Authors:** 


### THE CORSET A CAUSE OF CONSUMPTION.

The corset, with its inflexible stays and hour-glass shape, grasps the expanding lungs in their lower part like an iron vise and prevents their proper filling with air. The lungs are thus crowded up into the upper part of the chest, and pressed against the projecting edges of the first ribs, upon which they move to and fro with the act of breathing. The friction thus produced occasions a constant irritation of the upper portion of the lung, which induces a deposit of tuberculous matter, and the individual becomes a prey to that dread disease, consumption—a sacrifice to a practice as absurd as it is pernicious.

The lower part of the chest being narrowed, thus preventing proper expansion of the lungs, the amount of air inhaled is insufficient to properly purify the blood by removing from it the poisonous carbonic acid which gives to impure blood its dark color, and is so fatal to the life of all animals. In consequence of this defective purification of the blood, the whole body suffers. None of the tissues are properly kept in repair. They are all poisoned. Particles of gross, carbonaceous matter are deposited in the skin, causing it to lose its healthy color and acquire a dead, leathery appearance and a dusky hue. The delicate nerve tissues are poisoned, and the individual is tormented with "nerves," sleeplessness, and fits of melancholy. Continuous pressure upon these parts may cause such a degree of degeneration of the muscles of the chest as to seriously impair the breathing capacity. Unused muscles waste away, and when pressure is applied in addition, the wasting and degeneration become still more marked. This is exactly what happens with those who wear their clothing tight about the waist.—*Good Health.*

